# Supplementary material for: The interactome of intact mitochondria by cross-linking mass spectrometry provides evidence for coexisting respiratory supercomplexes
Source: Mol Cell Proteomics. 2017 Dec 8;17(2):216–32. doi: 10.1074/mcp.RA117.000470 (PMC5795388; doi:10.1074/mcp.RA117.000470)
Supplement: Supplemental Data [file supp_RA117.000470_133922_0_supp_23978_fzffwf.zip › spectra_annotation/mito_DR_spectra_annotation/365-1-5-1-62-1.pdf]

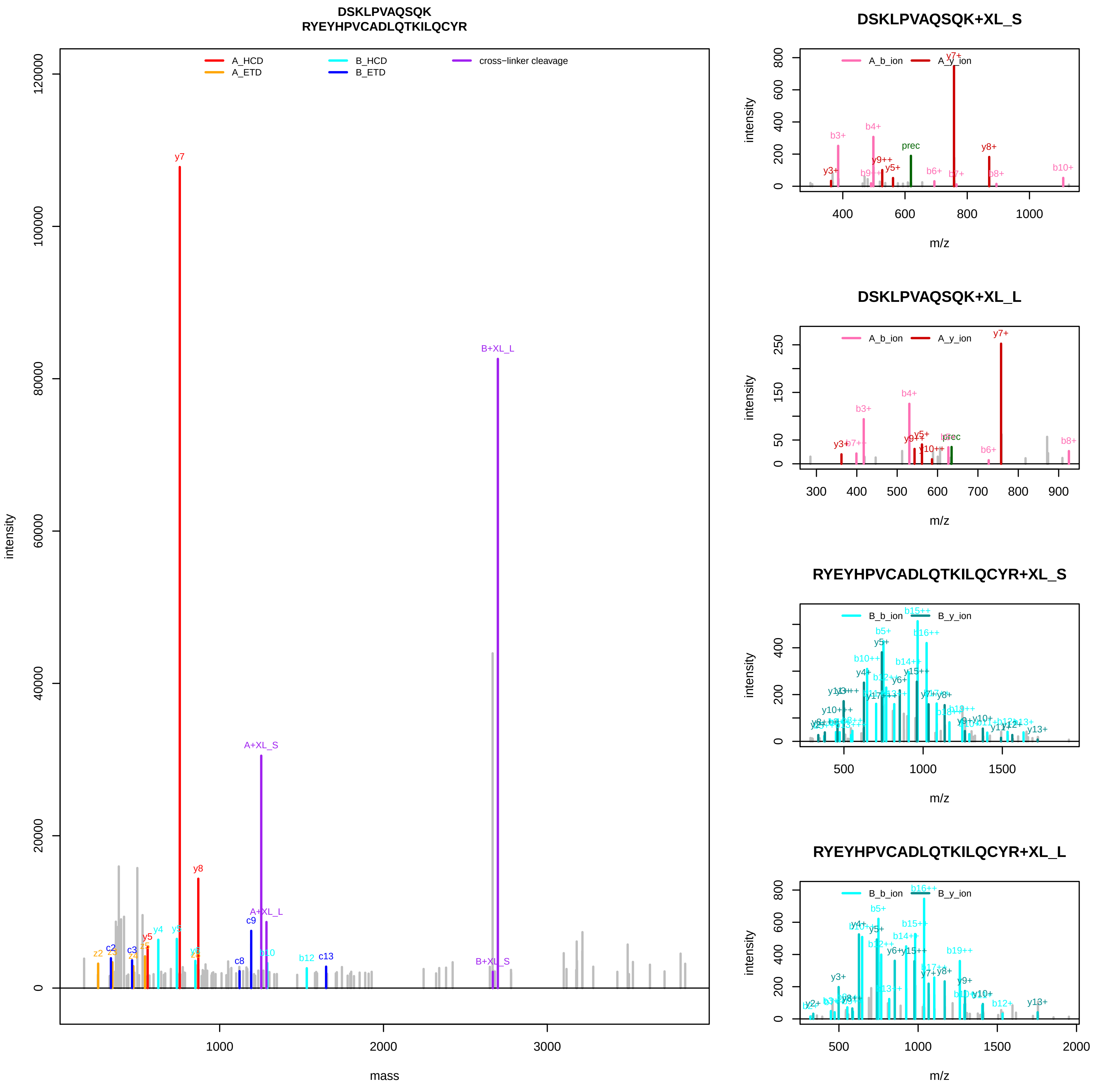
DSKLPVAQSQK
RYEYHPVCADLQTKILQCYR
A_HCD
A_ETD
B_HCD
B_ETD
cross−linker cleavage
intensity
mass
0
20000
40000
60000
80000
100000
120000
1000
2000
3000
y7
y8
y5
y4
B+XL_L
A+XL_S
A+XL_L
B+XL_S
c2
c3
c8
c9
c13
b12
z2
DSKLPVAQSQK+XL_S
A_b_ion
A_y_ion
y7+
y8+
b3+
b4+
prec
y3+
y9++
y5+
b6+
b10+
DSKLPVAQSQK+XL_L
b3+
b4+
b6+
b8+
y3+
y5+
RYEYHPVCADLQTKILQCYR+XL_S
B_b_ion
B_y_ion
b15++
b5+
b16++
y5+
b10++
b14++
y4+
y13+
RYEYHPVCADLQTKILQCYR+XL_L
B_b_ion
B_y_ion
b16++
b5+
y4+
b15++
b19++
y3+
y13+
m/z
intensity
